# A Denoising Scheme for Randomly Clustered Noise Removal in ICCD Sensing Image

**DOI:** 10.3390/s17020233

**Published:** 2017-01-26

**Authors:** Fei Wang, Yibin Wang, Meng Yang, Xuetao Zhang, Nanning Zheng

**Affiliations:** Institute of Artificial Intelligence and Robotics, Xi’an Jiaotong University, Xi’an 710049, China; wfx@xjtu.edu.cn (F.W.); wybxy001@126.com (Y.W.); xuetaozh@xjtu.edu.cn (X.Z.); nnzheng@xjtu.edu.cn (N.Z.)

**Keywords:** ICCD image sensor, low-light-level, sparse representation, randomly clustered noise, image denoising

## Abstract

An Intensified Charge-Coupled Device (ICCD) image is captured by the ICCD image sensor in extremely low-light conditions. Its noise has two distinctive characteristics. (a) Different from the independent identically distributed (*i.i.d.*) noise in natural image, the noise in the ICCD sensing image is spatially clustered, which induces unexpected structure information; (b) The pattern of the clustered noise is formed randomly. In this paper, we propose a denoising scheme to remove the randomly clustered noise in the ICCD sensing image. First, we decompose the image into non-overlapped patches and classify them into flat patches and structure patches according to if real structure information is included. Then, two denoising algorithms are designed for them, respectively. For each flat patch, we simulate multiple similar patches for it in pseudo-time domain and remove its noise by averaging all the simulated patches, considering that the structure information induced by the noise varies randomly over time. For each structure patch, we design a structure-preserved sparse coding algorithm to reconstruct the real structure information. It reconstructs each patch by describing it as a weighted summation of its neighboring patches and incorporating the weights into the sparse representation of the current patch. Based on all the reconstructed patches, we generate a reconstructed image. After that, we repeat the whole process by changing relevant parameters, considering that blocking artifacts exist in a single reconstructed image. Finally, we obtain the reconstructed image by merging all the generated images into one. Experiments are conducted on an ICCD sensing image dataset, which verifies its subjective performance in removing the randomly clustered noise and preserving the real structure information in the ICCD sensing image.

## 1. Introduction

**Figure 1:**
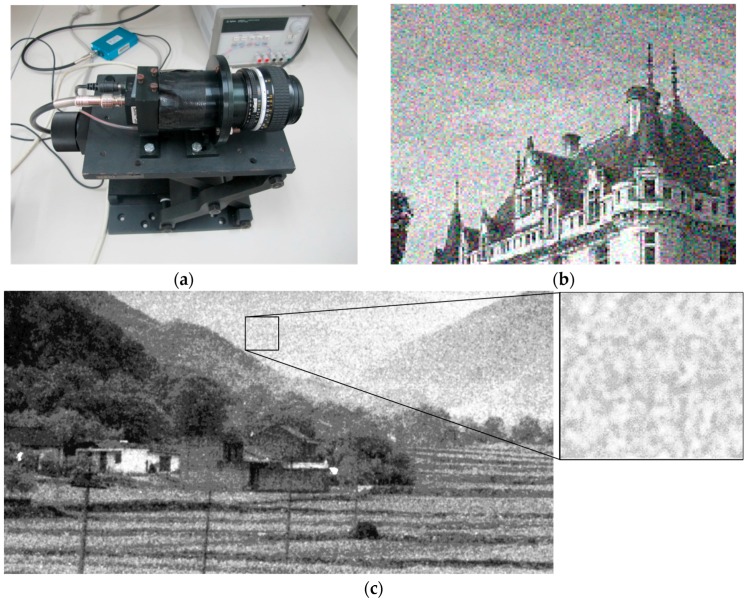
(**a**) The used ICCD device; (**b**) a natural image with white noise; (**c**) an image captured by ICCD image sensor (enhanced by histogram equalization) and its noise pattern.

An Intensified Charge-Coupled Device (ICCD) image sensor is used to capture the scene at extremely low-light-level conditions [1,2,3]. The principle of the ICCD image sensor is briefly described as follows. First, some electrons are obtained and injected into many voltages applied microchannel tubes after photovoltaic conversion process. Then, each electron crashes into the wall of a tube to generate more electrons. After that, all the electrons will be ejected from the tubes and shot onto the fluorescent screen. Finally, the image is captured by the CCD image sensor [4,5,6]. In this way, the ICCD image sensor could capture the scene in a low-light environment. Figure 1a shows the used device for ICCD image sensing in our project.

Although an ICCD image sensor has imaging ability in low-light environments, it may increase the noise significantly due to the randomly-generated electrons. So the noise in the ICCD sensing image may be much more complicated than that in the natural image. It has the following characteristics. (a) Different from the general independent identically distributed (*i.i.d.*) noise in natural image, the noise in the ICCD sensing image is spatially clustered due to the usage of microchannel tubes. It destroys the true structure information of the image and also induces unexpected structure information; (b) The noise in the ICCD image appears in a randomly clustered pattern. This is different from fixed pattern noise. In fixed pattern noise, the structure of the noise is often fixed or similar, like the raindrops in a natural image. Fixed pattern noise has been well studied in the literature [7,8,9,10,11]. By comparison, the structure of the noise in the ICCD image is random. We show an example in Figure 1 to illustrate the characteristics of the ICCD image. Figure 1c shows a captured ICCD image with the device in Figure 1a. It is seen that, the ICCD image includes unexpected structure information and its pattern appears random, which consequently destroys the true structure information of the image. By comparison, Figure 1b shows a natural image captured by a general CCD sensor, whose structure information is still kept intact despite the existence of the white noise. In this paper, we focus on this randomly clustered noise problem in the ICCD image. Indeed, this problem exists not only in the ICCD image but also other possible scenarios, like the image captured in front of a frosted window. Therefore, effective methods are required to remove such noise in the ICCD sensing image.

Image denoising has been well-studied in recent decades [12,13,14]. Generally, image denoising methods can be categorized into three classes: spatial-domain based methods, transform-domain based methods, and dictionary learning based methods [15].

Spatial-domain based methods mainly utilize the spatial correlations of natural images [16]. For each patch (or pixel), a set of candidates is often generated and used in the filtering process. According to the selection of candidates, spatial filters can be categorized as local and nonlocal filters. A large number of local filtering methods are designed, such as Wiener filter [17], least mean squares filter [18], trained filter (TF) [19], bilateral filter [20], anisotropic filtering [21], steering kernel regression (SKR) [22], and kernel-based image denoising employing semiparametric regularization (KSPR) [23]. However, these methods often require the signal-to-noise ratio to be low enough. Otherwise, the spatial correlation of neighboring pixels may be corrupted by severe noise. The nonlocal filters often make use of the self-similarity of different regions in natural images. For example, nonlocal means (NLM) method [24] filters a patch by weighted averaging all other patches in the image. Many methods have been proposed based on the NLM to reduce its computational cost [25,26] and enhance the denoising performance [27,28]. In recent years, some new solutions are also proposed for noise removal in the spatial-domain. Low rank matrix approximation (LRMA) is a newly emerging denoising technique, which recovered the underlying low rank matrix from its degraded observation. The authors of [29] proposed a model, named the ‘Weighted Schatten p-Norm Minimization (WSNM)’ model, to relax the nonconvex rank minimization problem for LRMA. In [30], they classified similar image patches to form multiple patch groups, then each group is factorized and estimated by singular value decomposition (SVD) separately. Similarly, Ref. [31] also classified similar image patches into groups, and each group is reconstructed separately using low-rank representation. For transform-domain based filtering methods, image patches are often represented by a set of coefficients based on orthonormal basis, such as wavelets [32], curvelets [33], and contourlets [34]. In these methods, small coefficients often represent the high-frequency part of the input image, which corresponds to image details and noise. Then the noise in the image could be eliminated by reducing the smaller coefficients. Plenty of transform-domain methods have been proposed in the past. For example, Ref. [35] used orthonormal basis for sharp frequency localization. In [36] they filtered an image in wavelet domain, in which the coefficients at adjacent positions and scales were firstly modeled as a Gaussian scale mixture model, then the wavelet coefficients were updated by Bayesian least-squares estimation. Besides, some methods incorporate nonlocal similarity into the transform-domain method [37,38,39]. For example, Ref. [39] proposed a block-matching and 3-D filtering (BM3D) method, which is based on [36]. The authors of [37] regularized each image patch by band-wise distribution modeling in transform domain. In [38], stripe noise is first separated using wavelet-Fourier filter, then remaining random noise is removed using the multiscale NLM filter. With the emergence of machine learning techniques in recent years, sparse representation has become a powerful method for image denoising and restoration problems. The general idea of this method is to learn a large group of patches from an image dataset to form a redundancy dictionary, such that each patch in the reconstructed image can be expressed as a linear combination of a few patches in the dictionary. There have been plenty of dictionary learning based denoising methods, such as K-clustering with singular value decomposition (K-SVD) [40], learned simultaneous sparse coding (LSSC) [41], clustering-based sparse representation (CSR) [42], hierarchical sparse learning with spectral-spatial information [43], and vector-valued sparse representation model using reduced quaternion matrix [44].

All the above denoising methods have been verified in removing the *i.i.d.* noise of natural images. However, they may not work well for an ICCD sensing image. For the spatial-domain based methods, they rely on spatial structure information of the image; however, the clustered noise in the ICCD sensing image includes unexpected structure information. For the transform-domain based methods, the clustered noise includes not only high-frequency information but also low-frequency information. So the transform-domain based methods could not eliminate the noise by reducing the high-frequency coefficients. For the dictionary learning based methods, they could not differentiate the unexpected structure information of the clustered noise in the ICCD sensing image, and attempt to recover it.

Besides the above methods for *i.i.d.* noise removal, there have been some methods for the clustered noise removal, such as water droplets or raindrops in the image. One dominant solution is called self-learning-based signal decomposition [9,10,11]. In this solution, the image is first decomposed into a low-frequency part and a high-frequency part, and a dictionary from the high-frequency part is learned. Then the feature of the noise pattern is extracted from the dictionary elements. After that, the dictionary is classified into noise-free sub-dictionary and noisy sub-dictionary by Support Vector Machine (SVM) [45] or clustering [46]. The noise-free sub-dictionary is used to reconstruct the high-frequency part by sparse representation. Finally, the image is recovered by merging the low-frequency part and the reconstructed high-frequency part. However, this solution is specifically designed for the clustered noise with fixed shape, like the water droplets or raindrops. It may not suit for an ICCD sensing image, in which the clustered noise is formed randomly. It is because: (a) the clustered noise in the ICCD sensing image exists not only in the high-frequency part, but also in the low-frequency part; (b) the noise pattern is so large that a single element of the dictionary cannot cover a clustered noise, and enlarging the size of the element may induce serious distortion; (c) the noise pattern is random, thus distinguishable features cannot be easily extracted.

In this paper, we specifically consider the randomly clustered noise problem of the ICCD image. We aim to remove not only the general noise in the ICCD image but also the additional structural information in it, which is induced by the randomly clustered noise. It may help improve the subjective quality of the ICCD image. We propose a denoising scheme to remove the randomly clustered noise in the ICCD sensing image. The basic idea of the scheme is to first divide the image into a flat region and a structure region to consider if real structure information is included, and then eliminate the noise in the flat region and reconstruct the structure information in the structure region. Specifically, we first segment the ICCD sensing image into non-overlapped patches. We design an identification feature based on Histogram of Oriented Gradient (HOG) [47] for this purpose, named Histogram Variance of Oriented Gradient (HVOG), which is used to decide whether a patch includes real structure information or not. With this feature, the patches can be classified into flat patches and structure patches. We then present two denoising algorithms for them separately. For each flat patch, we remove the randomly clustered noise in pseudo-time-domain, considering that unexpected structure information induced by the noise varies over time. It simulates multiple similar image patches for the patch in a period of time and eliminates the randomly clustered noise by averaging all the simulated patches. For each structure patch, we design a structure-preserved sparse coding method to restore the true structure information. It reconstructs each patch by describing it as a weighted summation of its neighboring patches and incorporating the weight information into the sparse representation process of the current patch.

By performing the above two algorithms for all flat patches and structure patches, respectively, we could get a whole reconstructed image. In the experiment, we find that blocking artifacts often exists in the reconstructed image, which is caused by the patch segmentation operation. To address this problem, we perform the above process iteratively by changing the relevant initialization parameters in the segmentation process and re-segmenting the ICCD sensing image into non-overlapped patches. Finally, we get the reconstructed ICCD sensing image by merging all the obtained images in the iterative process into one. The proposed denoising scheme is evaluated on a real ICCD sensing image dataset and compared with three existing denoising algorithms, which shows that the proposed scheme effectively removes the randomly clustered noise in the ICCD sensing image and also preserves the real structure information of the image simultaneously.

The rest of the paper is organized as follows. In Section 2 we introduce the framework of the proposed scheme. In Section 3, we present the scheme in details, including histogram variance of oriented gradient, denoising method in the pseudo-time domain, and structure-preserved sparse coding. In Section 4, experiments are performed for the proposed denoising scheme.

## 2. Framework of the Proposed Denoising Scheme for ICCD Sensing Image

**Figure 2:**
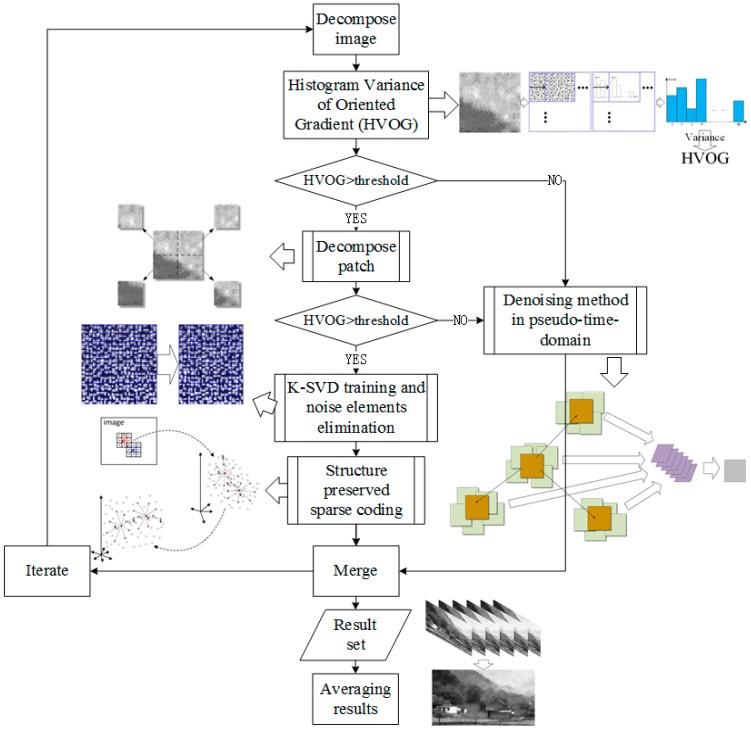
The framework of the denoising scheme for ICCD sensing image.

The framework of the proposed scheme is shown in Figure 2. First, we decompose an image into non-overlapped patches, then we design a feature HVOG to classify the patches into two classes based on a predefined threshold: structure patch and flat patch. For structure patches, we further decompose the patch into sub-patches and decide the type of each sub-patch by the feature HVOG. For the structure sub-patches, we design a structure-preserved sparse coding method to reconstruct the structure information. The relevant dictionary is initialized with the K-SVD dictionary trained from the structure sub-patches and its noise elements are eliminated. For the flat patches or the flat sub-patches, we design a denoising method in pseudo-time-domain. After all the patches and sub-patches are processed, they are merged into one image. The above process is repeated multiple times by changing the initialization parameters. Finally, we get the reconstructed ICCD sensing image by merging all the results in each iteration process.

## 3. The Denoising Scheme for ICCD Sensing Image

In this section, we present the proposed denoising scheme in detail, including four parts, i.e., the histogram variance of oriented gradient feature, the denoising method in pseudo-time-domain, the structure-preserved sparse coding, and the iterative process. Considering that the captured image by the ICCD image sensor is often of low contrast, we enhance the input image with histogram equalization before the denoising process.

### 3.1. Patch Classification Based on Histogram Variance of Oriented Gradient

In this section, we first segment the ICCD image into non-overlapped patches, and then divide them into two parts—i.e., flat patches and structure patches—according to if true structure information of the scene is included. Generally, the flat patches and the structure patches in the ICCD sensing image are different from that in nature image due to its distinctive characteristics. For the flat patches in the ICCD sensing image, it mainly includes the clustered noise, and the pattern of the clustered noise is formed randomly. In this scenario, the feature of the flat patches may also be distributed randomly, e.g., the magnitudes and orientations of the gradient feature. However, for the structure patches, they include not only the clustered noise but also some texture information. Different from the clustered noise, the gradient feature of the texture information may not distribute randomly. For example, the orientations of the gradient feature of a patch near a straight edge may be the same.

Based on the above analysis, we design a feature based on HOG [47], named HVOG, to differentiate the flat patches from the structure patches based on the magnitudes and orientations of the gradient feature. Generally, the magnitudes and orientations of the gradient in a flat patch are often random due to the randomness of the clustered noise in the ICCD sensing image. So the distribution of the gradient feature may be uniform in a flat patch. However, it may not be uniform in a structure patch due to the existence of the texture information.

**Figure 3:**
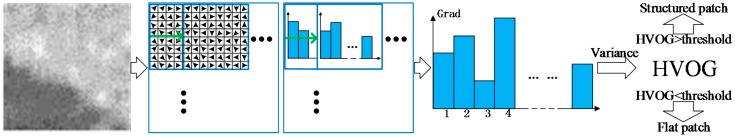
The patch classification process.

The patch classification process is as follows (see Figure 3):

(1) Slide a window of size *s* × *s* over each patch with step *t*, to obtain multiple blocks, calculate the magnitudes and orientations of the gradient feature in each block.
(2) Divide the pixels in each block into *k* classes according to their orientations, then sum up the absolute magnitudes of the gradients of all pixels in each class to form a histogram with the results of all *k* classes.
(3) Repeat the process above to form histograms for all blocks, then merge these histograms into one in the series.
(4) Calculate the variance of the merged histogram.
(5) Predefine a threshold value. If the variance is larger than the threshold, the patch is classified as a structure patch, otherwise, it is classified as a flat patch.

With the above method, the whole ICCD sensing image can be segmented and classified into flat patches and structure patches. The parameters *s*, *t*, *k*, and the threshold will be determined by experiment in Section 4.1.

### 3.2. Denoising Method of Flat Patches in Pseudo-Time Domain

In last section, the patches are classified into flat patches and structure patches. For each flat patch, it often includes some unexpected structure information due to the existence of the clustered noise. General spatial filters do not work in this scenario, like the Gaussian filter. We address this problem in time domain by averaging images of the same scene captured in a period of time, because the structure information of the clustered noise varies along time. The problem is that we only have one image, not a video. To address this problem, we present a method to simulate the images of different times based on the local information of one image. The method is described as follows.

First, we select two kinds of patches in the image, named neighbor patches and block-matching patches, as follows.

**Neighbor patches.** We select some neighbor patches that are adjacent to current patch. They should meet the following limitations: (1) the distance between the centers of current patch and an adjacent patch are less than half the length of the patch; (2) the HVOG value of the patch should be less than a predefined threshold (the same threshold as that of in Section 3.1), which ensures the patch is a flat one; (3) the average gray value between an adjacent patch and its reference patch is less than a predefined threshold value (fixed as 0.005 in this paper). It ensures they have similar gray values.

**Block-matching patches.** We further select some patches in the image as block-matching patches, who have similar contents with the current patch. First, we calculate the residues between each patch and the current patch. Then the residue is compared with a predefined threshold (fixed as 0.0015 in this paper). These patches with smaller residues are selected as the block-matching patches.

Then, we remove the randomly clustered noise in each flat patch by averaging all the obtained neighbor patches and block-matching patches.

### 3.3. Denoising Method of Structure Patches Based on Its Sparse Representation

Considering that a structure patch may still contain small flat regions, we further decompose each structure patch into sub-patches and classify these sub-patches as flat sub-patches and structure sub-patches with the method in Section 3.1. For the flat sub-patches, they are also denoised with the method in Section 3.2. For structure sub-patches, they are denoised as follows.

We develop a method based on sparse representation to reduce the clustered noise in the structure sub-patches. First, we train a K-SVD dictionary based on all the structure sub-patches and eliminate its noise elements. Then, we design a structure-preserved sparse coding method to restore the structure information. It reconstructs each patch by first describing it as a weighted summation of its neighboring patches and then incorporating the weight information into the sparse representation process of the current patch. Finally, we could get the reconstructed patch for the current patch. The method is described as follows.

#### 3.3.1. K-SVD Training and Noise Elements Elimination

We assume there are totally *N* structure sub-patches for training. They are denoted as $y_{i}\in {\mathbb{R}}^{n}$ (1≤i≤N) and $Y={\left\{y_{i}\right\}}_{i=1}^{N}$. In the sparse representation of each signal $y_{i}$, an over-complete dictionary matrix $D\in {\mathbb{R}}^{n\times K}$ is first trained that contains *K* prototype signal-atoms for columns, then the signal can be represented as a sparse linear combination of these elements as $y_{i}\approx Dx_{i}$. It satisfies $∥y_{i}-Dx_{i}{∥}_{p}\leq \epsilon$ and $x_{i}\in {\mathbb{R}}^{K}$ denotes the representation coefficients of the signal $y_{i}$, where $∥\cdot {∥}_{p}$ is the $l^{p}$ norm (*p* = 1, 2, or ∞). The sparse representation problem can be solved with the following objective function based on the K-SVD [40]:

(1) $$\underset{D,X}{\min}∥Y-DX{∥}_{F}^{2}\;s.t.\;\forall i,\;∥x_{i}{∥}_{0}\leq T_{0}$$

where $∥Y-DX{∥}_{F}^{2}=\sum_{i=1}^{N}∥y_{i}-Dx_{i}{∥}^{2}$, $∥\cdot {∥}_{0}$ is the $l^{0}$ norm, $X={\left\{x_{i}\right\}}_{i=1}^{N}$, and $T_{0}$ is a predetermined value fixed as 3 in this paper following the suggestion in [40].

After that, we eliminate the noise elements in the dictionary. First, we reshape the elements of the dictionary into square matrixes like a set of image patches. Second, we obtain the HVOG values of these matrixes with the method in Section 3.1. Then the matrixes with small HVOG values are dropped and others are reserved as a new dictionary. Finally, the new dictionary is used to obtain the coefficients by applying the OMP [48] method and the coefficients are used for initialization of sparse coding later.

#### 3.3.2. Structure-Preserved Sparse Coding

We present a method to preserve the structure information based on sparse coding by adding a regularization term into the objective function in Equation (1), which was also studied in [49,50,51]. In this method, the structure relationship among patches in a local region is often encoded into the sparse codes. We take the manifold structure of local patches as the regularization term as follows:

(2) $$\underset{D,X}{\min}\overset{N}{\underset{i=1}{\sum }}∥y_{i}-Dx_{i}{∥}^{2}+\beta \overset{N}{\underset{i=1}{\sum }}{∥x_{i}-\sum_{j}w_{i,j}x_{i,j}∥}^{2}\;s.t.\;\forall i,∥x_{i}{∥}_{0}\leq T_{0}$$

In Equation (2), β is a constant parameter and its value will be determined in Section 4.2.1, $x_{i,j}$ denotes the representation coefficient of the *j*-th neighbor sub-patch of the *i*-th sub-patch, and $W_{i}={\left\{w_{i,j}\right\}}_{j=1}^{8}$ denotes the local manifold structure vector, which is determined as follows.

The local manifold structure vector $W_{i}$ in Equation (2) is used to represent the correlation of the structure information between the *i*-th sub-patch and its eight neighbor sub-patches with sparse coding. We approximately determine it in spatial domain, considering that the correlation of the structure information of different patches in the spatial domain may be similar to that with sparse coding. First, we calculate the average gray values of the *i*-th sub-patch and its eight neighbors, denoted $p_{i,0}$ and $p_{i,j}$ (1≤j≤8). Then we determine the vector $W_{i}$ by solving the least squares estimation problem as follows:

(3) $$W_{i}=\arg\underset{W_{i}}{\min}{\left(p_{i,0}-\overset{8}{\underset{j=1}{\sum }}w_{i,j}p_{i,j}\right)}^{2}$$

Note that, we use the average gray value of each block rather than the content of the block to alleviate the effect of the noise in the ICCD sensing image.

Then the optimization problem of Equation (2) is solved as follows:

• Step (a): calculate the sparse coefficients X by fixing the dictionary D.

The dictionary D is fixed as the previously obtained new dictionary in Section 3.3.1. In this scenario, the objective function in Equation (2) is a convex. We solve the minimization problem of Equation (2) with gradient descent method. First, we calculate the gradient of the objective function in Equation (2) with respect to $x_{i}$ as follows:

(4) $$grad\left(x_{i}\right)=\;\frac{\partial }{\partial x_{i}}\left(∥y_{i}-Dx_{i}{∥}^{2}+\beta {∥x_{i}-\sum_{j}w_{i,j}x_{i,j}∥}^{2}\right)\;=\;2\left(\left(D^{T}D+\beta \right)x_{i}-D^{T}y_{i}-\beta C_{i}\right)$$

where $C_{i}=\sum_{j}w_{i,j}x_{i,j}$. Then the sparse coefficients can be obtained with gradient descent method as follows:

(5) $$x_{i}^{\left(n+1\right)}=\theta \left(x_{i}^{\left(n\right)}-\frac{\eta }{2}\cdot grad\left(x_{i}^{\left(n\right)}\right)\right)\;=\theta \left(x_{i}^{\left(n\right)}-\eta \left(\left(D^{T}D+\beta \right)x_{i}^{\left(n\right)}-D^{T}y_{i}-\beta C_{i}\right)\right)$$

where (n), denotes the *n*-th iteration of the process, $x_{i}^{\left(0\right)}$ is initialized as the coefficients obtained with the new dictionary in Section 3.3.1, and η denotes step length (fixed as 0.4 in this paper). θ(·) is the function that only reserves the $T_{0}$ largest absolute values of the vector. It is used to meet the constraint $∥x_{i}{∥}_{0}\leq T_{0}$ in each iteration of the process. We finally obtain the sparse coefficient $X={\left\{x_{i}\right\}}_{i=1}^{N}$.

• Step (b): calculate the dictionary D by fixing the sparse coefficients X.

After $X={\left\{x_{i}\right\}}_{i=1}^{N}$ is obtained in step (a), we try to obtain D in the optimization problem of Equation (2). Similar problems have been well-studied in the literature like the Maximum Likelihood Method [52] and the Maximum A-Posteriori Probability method [53,54,55,56]. In this paper, we adopt the latter method. Then the dictionary D can be obtained with the following iterative process:

(6) $$D^{\left(n+1\right)}=D^{n}+\eta^{\prime }EX^{T}+\eta^{\prime }\cdot tr\left(XE^{T}D^{\left(n\right)}\right)D^{\left(n\right)}$$

where $E=\left[e_{1},e_{2},\;\ldots ,e_{N}\right]$, $e_{i}=y_{i}-D^{\left(n\right)}x_{i}+\beta \cdot D^{\left(n\right)}\left(C_{i}-x_{i}\right)$, $\eta^{\prime }$ is the step length of the iterative process (fixed as 0.2 in this paper), β is the same as in Equation (2), $D^{\left(n\right)}$ is initialized as the new dictionary obtained in Section 3.3.1.

Finally, iterate the above Steps (a) and (b) several times. The number of iterations is generally set as three in this paper following the suggestion in [49].

After the dictionary D and coefficients X are obtained, we reconstruct the *i*-th structure sub-patch of the ICCD sensing image as follows:

(7) $$IM_{i}=\arg\underset{IM_{i}}{\min}\delta ∥y_{i}-IM_{i}{∥}^{2}+∥Dx_{i}-IM_{i}{∥}^{2}$$

where $IM_{i}$ denotes the column form of the sub-patch, and δ is a constant parameter (0.1 in this paper). Its solution is:

(8) $$IM_{i}=\frac{\delta \cdot y_{i}+Dx_{i}}{1+\delta }$$

With Equation (8), all *N* structure sub-patches can be reconstructed.

### 3.4. Iterative Process

With the methods in Section 3.2 and Section 3.3, we could reconstruct all flat patches (sub-patches) and structure sub-patches. Then we could obtain the whole reconstructed image by combining them. However, in the experiment, we find that blocking artifacts often appear in the reconstructed image. It is possibly because the image is segmented as non-overlapped patches and they are processed separately. To address this problem, we iteratively perform the reconstruction process by changing relevant parameter value in the segmentation process. Specifically, we change the start point in the image. In this manner, we finally obtain the reconstructed image by averaging all the reconstructed images in the iterative process. Ideally, the iteration number of the procedure is determined by the number of possible start points in the image. For example, there are 1024 possible start points if the size of the patch is 32 × 32 pixels. However, repeating the procedure too many times will significantly increase the computational cost. To address this problem, we change the start point every 4 × 4 pixels in the experiment. In this manner, the iterative process is reduced as 64 in our solution.

## 4. Experiments and Analysis

In this section, we firstly determine the relevant parameters in the patch classification method in Section 3.1 by experiment, then test the performance of the structure-preserved sparse coding method in Section 3.3. Finally, we verify the performance of the proposed denoising scheme in real ICCD sensing images and compare with three methods.

### 4.1. Parameter Determination in the Patch Classification Method

In the proposed patch classification method, there are three relevant parameters: the size of the block *s* × *s*, the step of the sliding window *t*, and the number of the orientation classes *k*. We determine these parameters by experiment. A real ICCD sensing image of size 6240 × 2005 pixels is used in the experiment. First, we segment the ICCD sensing image into 64 × 64 patches, and manually classify some of them into flat patches and structure patches. Then we perform the proposed patch classification method on the same ICCD sensing image to obtain the HVOG values of all patches, which are used for patch classification in the method. We calculate the average HVOG values of the flat patches and structure patches, and denoted $F_{HVOG}$ and $S_{HVOG}$, respectively. We define the following cost to evaluate the performance:

(9) $$DS=\frac{S_{HVOG}-F_{HVOG}}{S_{HVOG}+F_{HVOG}}$$

Generally, larger *DS* value indicates that the patches can be well classified with their HVOG values. Table 1 shows the experimental results with different parameter values.

**Figure 4:**
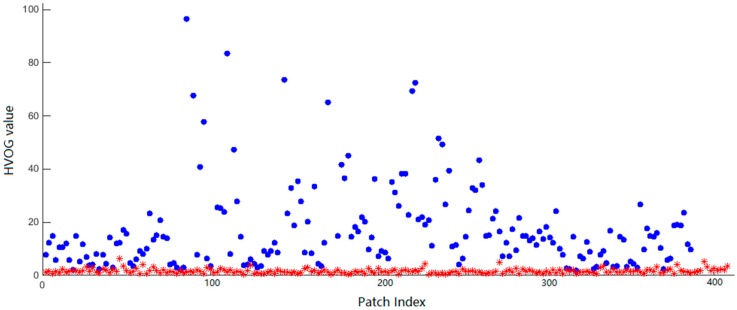
The obtained HVOG values (multiplied by 1000) of all patches. The HVOG values of the structure and flat patches are shown in blue and red, respectively.

We see that the *DS* value increases with the decreasing *k* value. For different *k* values, the *DS* value is always maximal when *t* = 16, *s* = 32. So we set the parameters as *k* = 3, *t* = 16, and *s* = 32 in our method. With these parameters, we obtain the HVOG values of all the patches and show them in Figure 4. It is seen that the HVOG values with the proposed method effectively classify the patches into structure patches and flat patches.

After the parameters *s*, *t*, and *k* are determined, we use these parameters to calculate the relative optimal value of the threshold. Table 2 shows the classification precision under different threshold. It is seen that the classification precision is maximal when threshold equals 0.0024. Based on this experiment, we set the threshold as 0.0024 in this paper.

### 4.2. Verification of the Structure-Preserved Sparse Coding in a Dataset with Simulated Noise

The proposed structure-preserved sparse coding method is the key point in denoising the ICCD sensing image. It is because the structure patches are hardly to be reconstructed compared with flat patches due to the existence of both the real structure information and the wrong one. The proposed method aims to remove the clustered noise while preserving the real structure information, which is developed based on sparse coding method. In order to effectively verify its performance in preserving the structure information, we test it separately and compare with general sparse coding methods. Rather than testing with real ICCD sensing image, we test its performance in a dataset with simulated clustered noise to clearly show its performance. The dataset is generated by randomly imposing clustered noise of different shapes and sizes in eight natural images. Then the proposed method is performed in this dataset for noise removal. In the experiment, the patch size is set as 8 × 8 pixels. Three general sparse coding methods are used for comparison, including DCT [57], K-SVD [40], and Beta Process (BP) [58]. These methods are used for denoising by replacing the trained dictionary and following the same method OMP [48].

#### 4.2.1. Parameter Determination in the Structure-Preserved Sparse Coding

**Figure 5:**
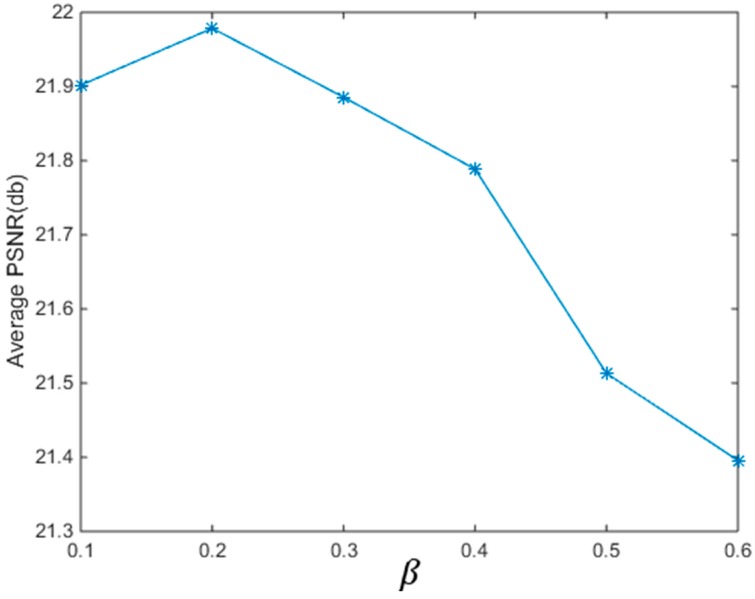
The average PSNR values with respect to the parameter β.

We obtain the parameter β in the Structure-Preserved Sparse Coding by training on the above dataset. We run the Structure-Preserved Sparse Coding with different parameter β and calculate the PSNR of each image. The PSNR values of each image under different β are concluded in Table 3 and the average results are shown in Figure 5. From Figure 5, we see that the average PSNR value is maximal at β=0.2. Based on this experiment, we set the parameter β as 0.2 in this paper.

#### 4.2.2. Results and Comparison

**Figure 6:**
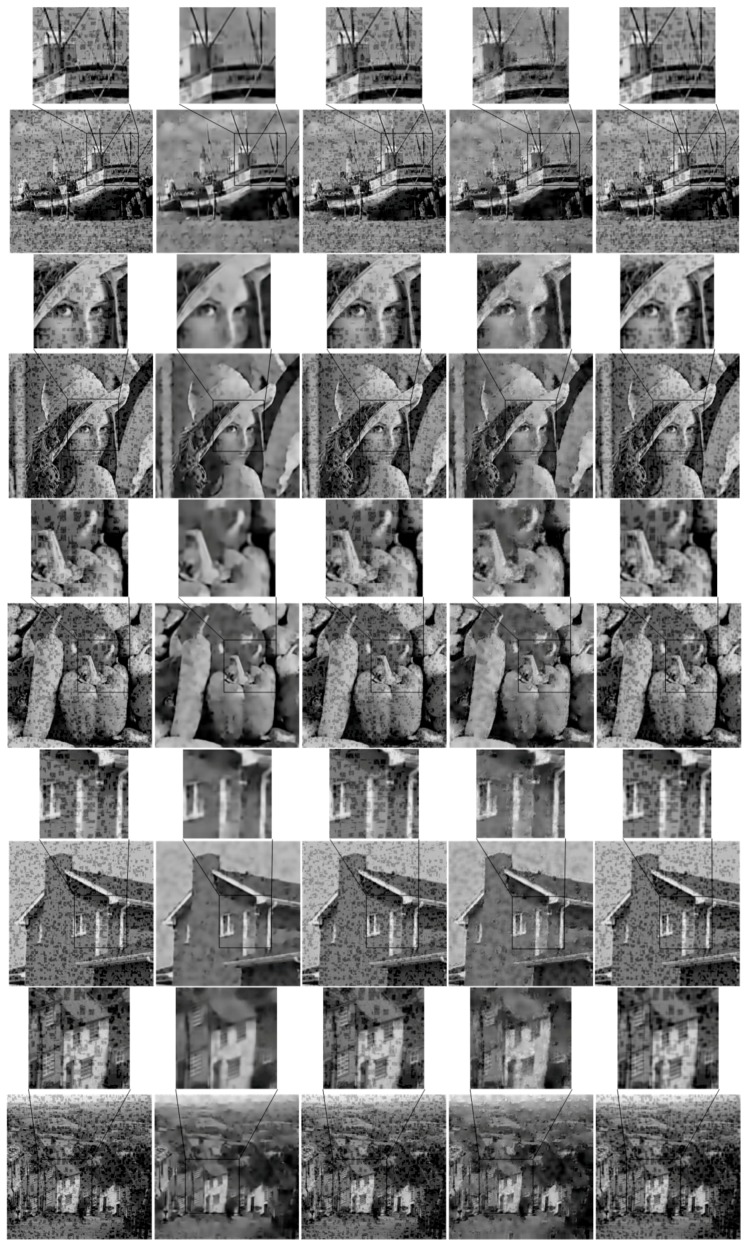

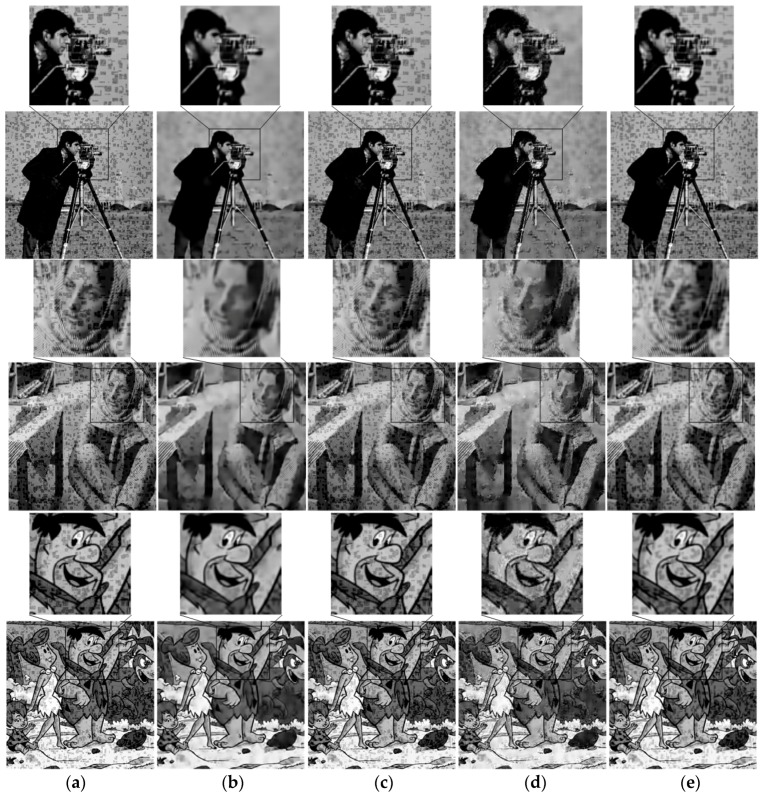
The subjective performance of the proposed structure-preserved sparse coding method: (**a**) the dataset with simulated clustered noise; (**b**) the results of proposed method; (**c**) the results of the method based on DCT; (**d**) the result of the method based on K-SVD; (**e**) the result of the method based on BP.

Figure 6 shows the subjective results of these methods. It is seen that the proposed method well removes the added clustered noise and preserves its own structure information simultaneously. By comparison, the denoising methods based on the DCT [57] and the BP [58] do not effectively remove the additional clustered noise, because they could not differentiate the clustered noise from its own structure information. The denoising method based on the K-SVD [40] could partly remove the clustered noise when the relevant parameter was properly chosen. However, it induces significant distortion in structure areas in this scenario.

Besides the subjective performance, we further tested the objective performance of the proposed solution. Table 4 further shows the objective results (in PSNR) of these methods. It is seen that the PSNR of the proposed solution is a little better than or comparable to others. Note that, the main purpose of this paper is to reduce the additional structural information caused by the randomly clustered noise, which helps improve its subjective performance.

### 4.3. Verification of the Proposed Scheme on Real ICCD Sensing Images

**Figure 7:**
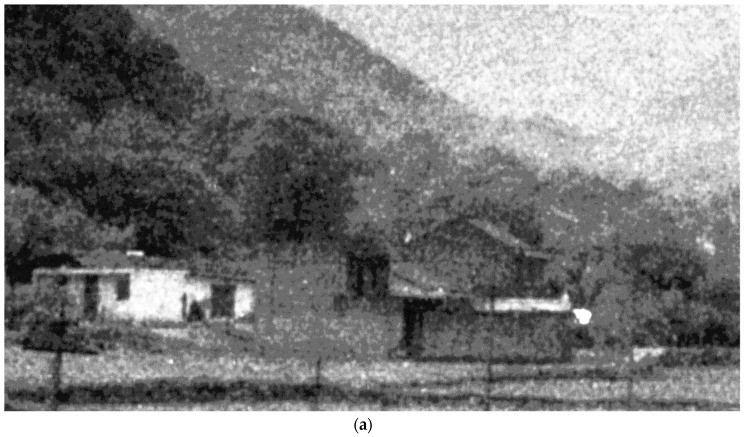

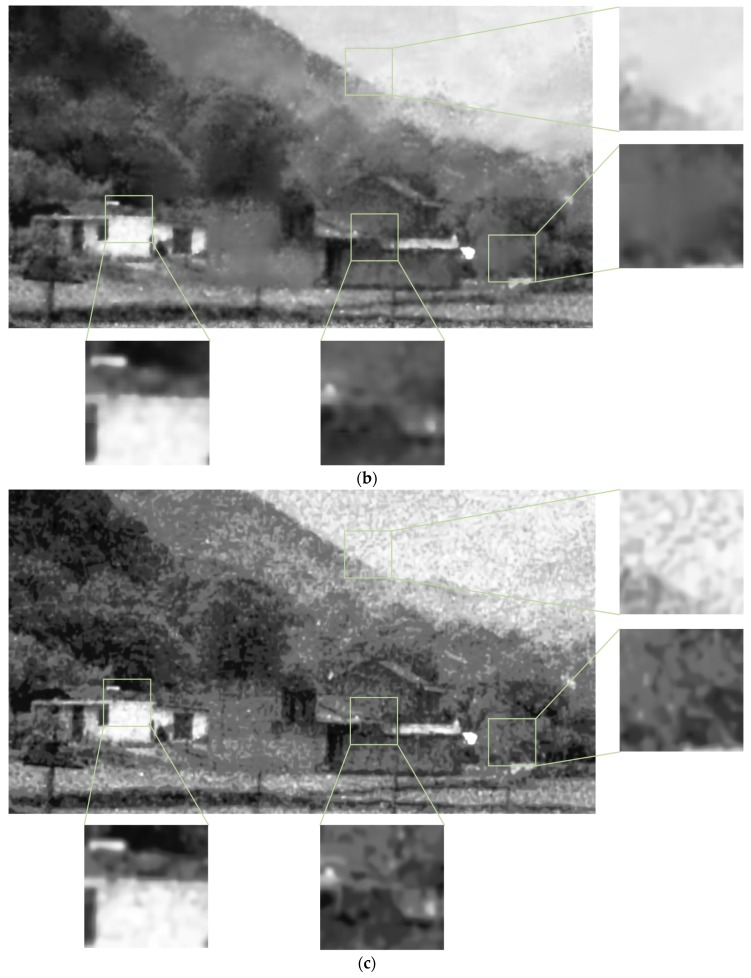

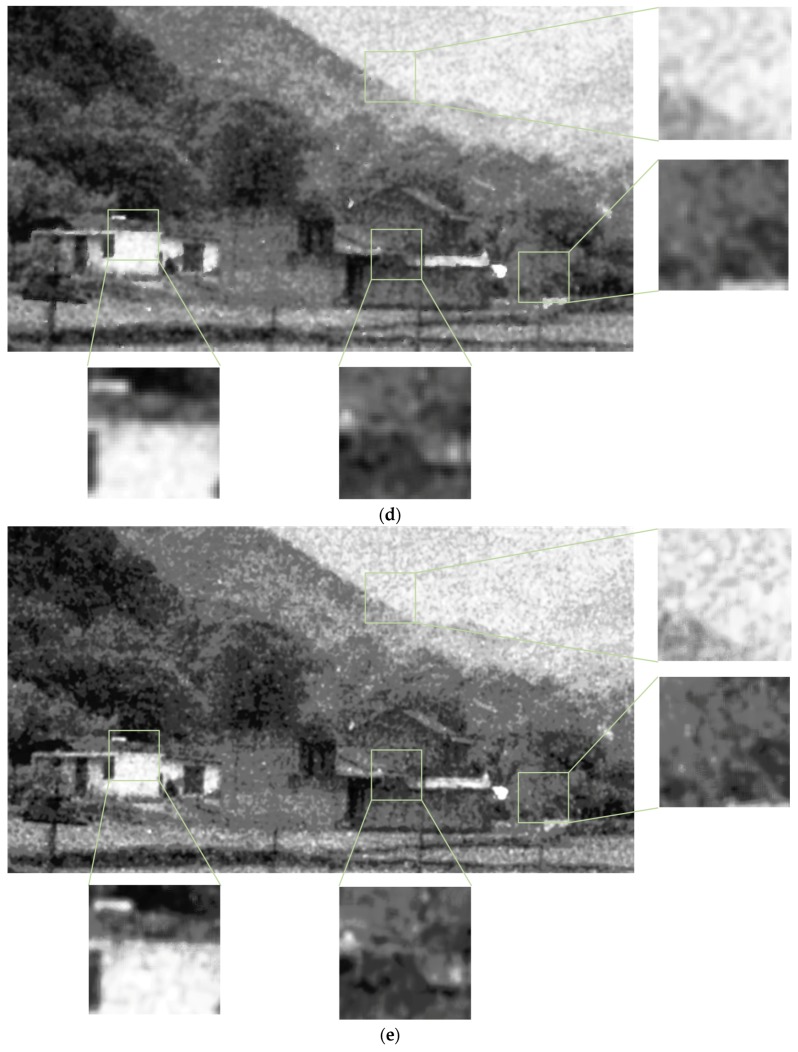
The subjective results of the proposed scheme and others. (**a**) a real ICCD sensing image; (**b**) the result of the proposed scheme; (**c**) the result of BM3D; (**d**) the result of the denoising method based on the K-SVD; (**e**) the result of BLS-GSM.

**Figure 8:**
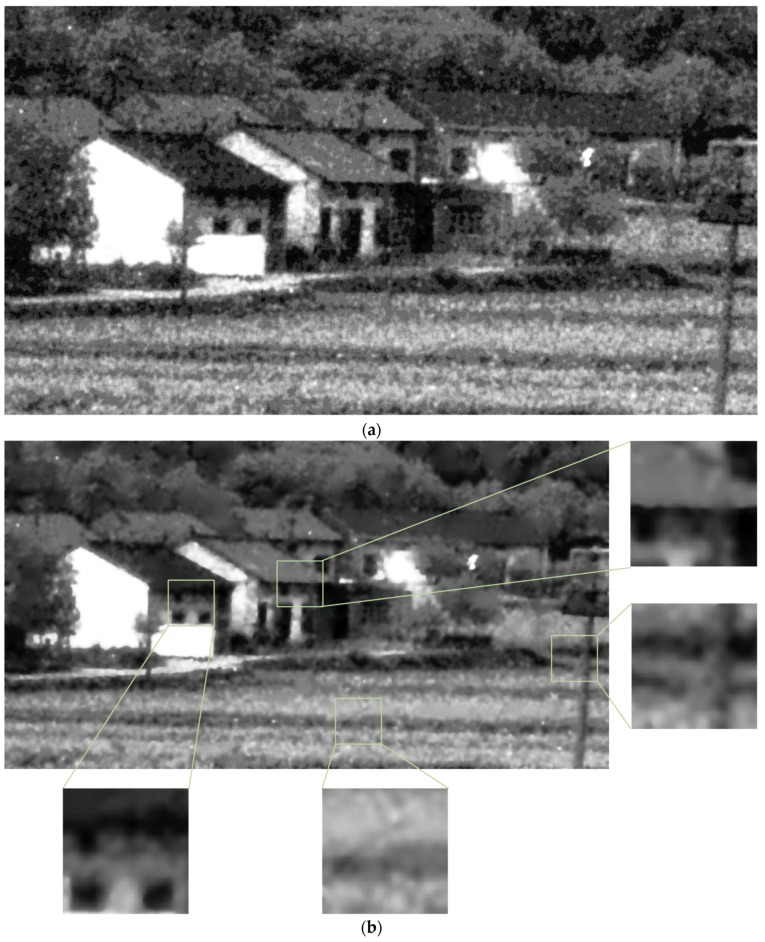

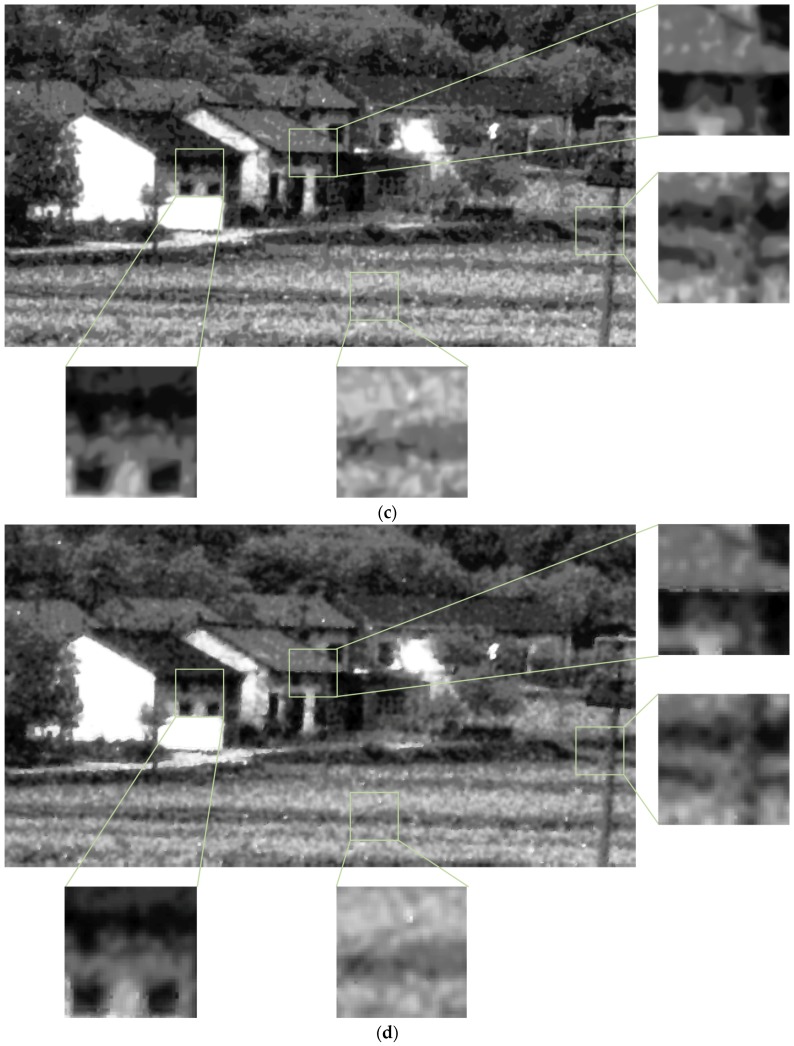

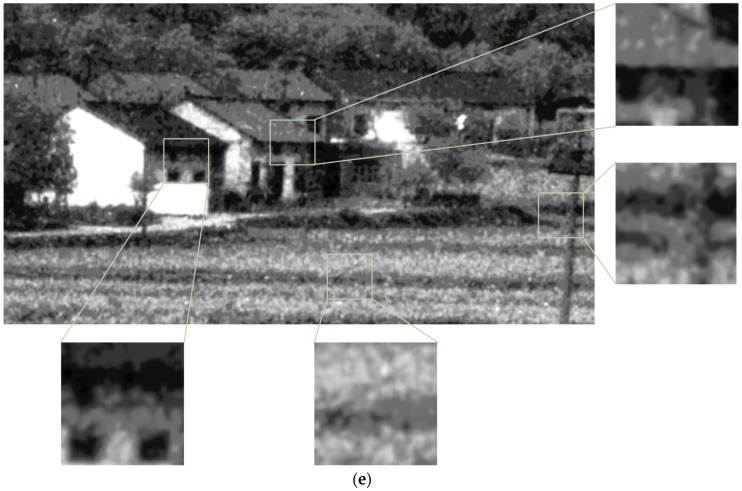
The subjective results of the proposed scheme and others. (**a**) a real ICCD sensing image; (**b**) the result of the proposed scheme; (**c**) the result of BM3D; (**d**) the result of the denoising method based on the K-SVD; (**e**) the result of BLS-GSM.

**Figure 9:**
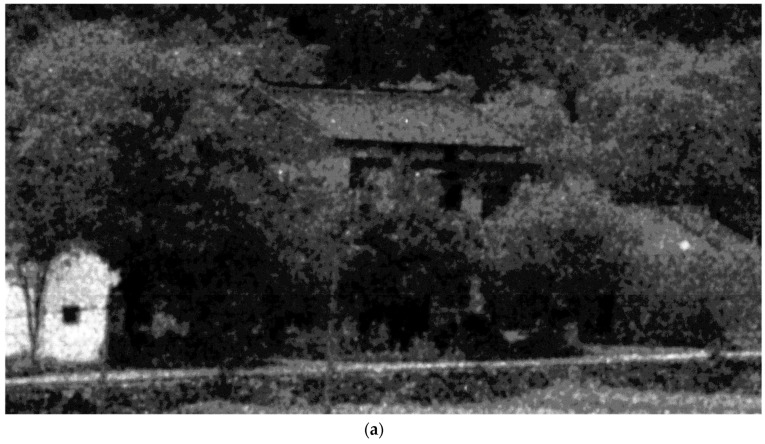

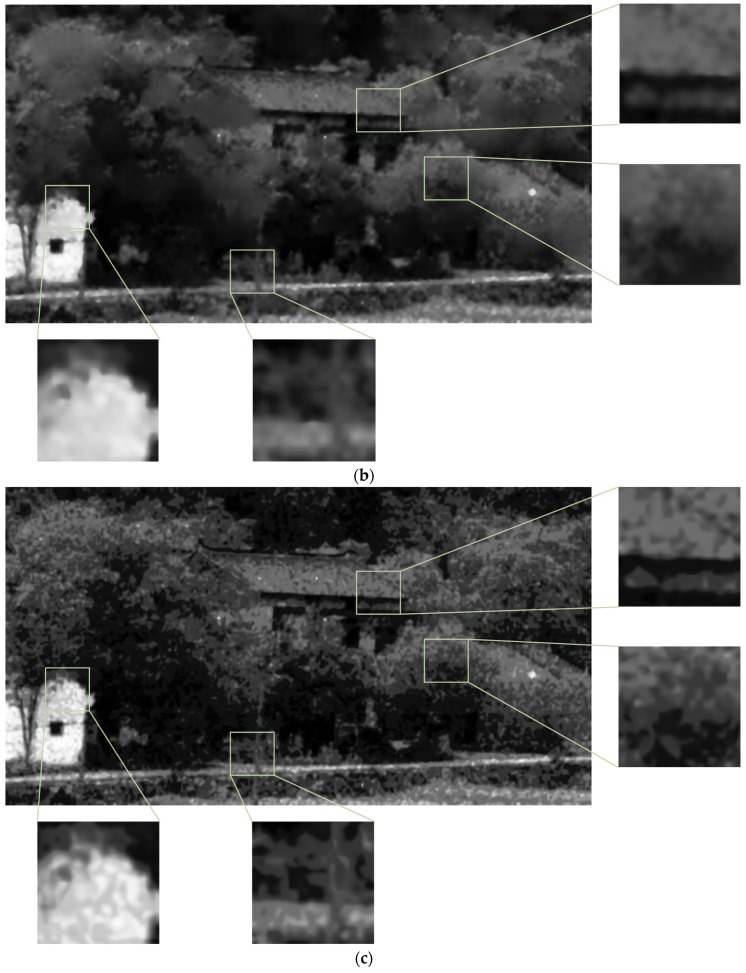

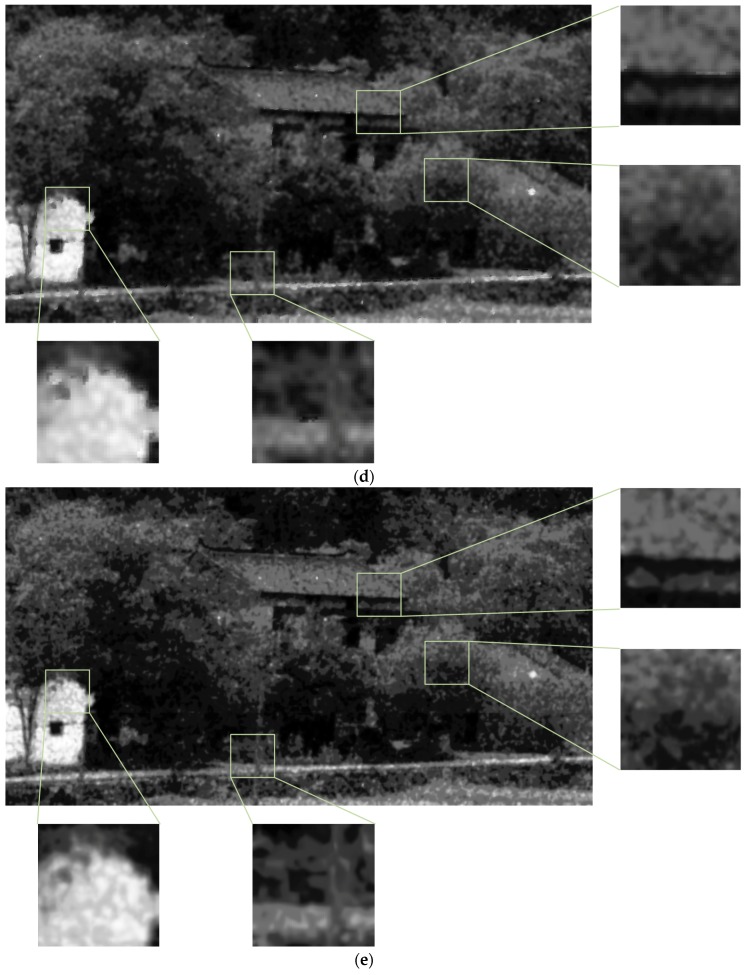
The subjective results of the proposed scheme and others. (**a**) a real ICCD sensing image; (**b**) the result of the proposed scheme; (**c**) the result of BM3D; (**d**) the result of the denoising method based on the K-SVD; (**e**) the result of BLS-GSM.

In this section, we test the performance of the proposed denoising scheme in real ICCD sensing images (Figure 7a, Figure 8a and Figure 9a). Note that, these test images have been enhanced with histogram equalization. To our knowledge, there are few denoising methods that consider clustered noise in the literature. So we mainly compare with three existing denoising methods, including the BM3D denoising method [39], K-SVD based denoising method [40], and the BLS-GSM denoising method [36]. Since the ground truth of the test images is not available, we mainly show the subjective results in this test.

It is seen in Figure 7, Figure 8 and Figure 9 that the proposed scheme effectively removes the clustered noise in not only the flat regions but also structure regions, meanwhile, some structure information is considered true and they are preserved. By comparison, the BM3D denoising method [39] and the BLS-GSM denoising method [36] could effectively remove the *i.i.d.* noise in the image, however, they do not work well in removing the clustered noise. More seriously, some clustered noise is even enhanced. It is because these methods often wrongly consider the clustered noise as true structure information in the denoising process. The K-SVD based denoising method [40] could partly remove the clustered noise in the ICCD sensing image when the relevant parameter is properly chosen, however, it may also induce significant distortion in structure areas. This is similar to the results in Section 4.2.2.

## 5. Conclusions

In this paper, we addressed the denoising problem of the image captured by the ICCD image sensor, in which the noise appears in a randomly clustered pattern. We designed a denoising scheme to remove the clustered noise and preserve its own structure information in the ICCD sensing image. Experimental results demonstrated the effectiveness of the proposed scheme by comparing it with three existing denoising methods.

## Figures and Tables

**Table 1:** The calculated *DS* values at different parameter values.

k	t	s	$S_{HVOG}$	$F_{HVOG}$	DS
3	8	16	0.0339	0.0079	0.6220
3	16	16	0.0359	0.0082	0.6281
3	16	32	0.0205	0.0017	0.8468
3	32	32	0.0256	0.0025	0.8221
4	8	16	0.0259	0.0082	0.5191
4	16	16	0.0306	0.0082	0.5773
4	16	32	0.0171	0.0018	0.8095
4	32	32	0.0196	0.0023	0.7900
5	8	16	0.0279	0.0080	0.5443
5	16	16	0.0289	0.0081	0.5622
5	16	32	0.0166	0.0018	0.8043
5	32	32	0.0180	0.0023	0.7334
6	8	16	0.0250	0.0077	0.5290
6	16	16	0.0259	0.0078	0.5371
6	16	32	0.0148	0.0018	0.7831
6	32	32	0.0159	0.0022	0.7569

**Table 2:** The classification precision under different thresholds.

**Threshold**	0.0020	0.0021	0.0022	0.0023	0.0024	0.0025	0.0026	0.0027
**Classification precision (%)**	93.20	93.45	94.17	94.90	95.39	94.42	93.32	92.72

**Table 3:** Objective result (in PSNR, dB) of each image under a different β.

	β=0.1	β=0.2	β=0.3	β=0.4	β=0.5	β=0.6
Boat	21.4533	21.6566	21.4246	21.2539	20.7531	20.5860
Lena	22.1502	22.2179	22.1357	21.8246	21.3215	21.1851
Peppers	22.1224	22.1707	22.1109	22.0165	21.9634	21.7340
House	22.7045	22.7334	22.7110	22.7031	22.6931	22.6879
Goldhill	21.9759	22.0216	22.0093	21.9678	21.8985	21.8648
Cameraman	22.2736	22.3714	22.2549	22.1832	22.0906	22.0154
Barbara	21.4946	21.6002	21.5034	21.3674	20.9760	20.8354
Flinstones	21.0398	21.0561	20.9341	20.7524	20.4086	20.2569
Average	21.9018	21.9785	21.8855	21.7886	21.5131	21.3957

**Table 4:** Objective result comparison (in PSNR, dB) of the proposed method with others.

	Noisy Image	Proposed	DCT	K-SVD	BP
Boat	19.5975	21.6566	20.3065	19.4485	20.5620
Lena	19.4215	22.2179	20.1788	19.9263	20.6286
Peppers	19.2094	22.1707	19.9566	20.0065	20.3398
House	18.9383	22.7334	19.7697	21.0326	20.1685
Goldhill	19.0536	22.0216	19.7694	19.4678	20.0385
Cameraman	19.8617	22.3714	20.6232	20.4032	20.8906
Barbara	19.7508	21.6002	20.4688	18.9671	20.8770
Flinstones	19.7653	21.0561	20.1872	17.7524	20.0086
